# The Naked Truth: The Face and Body Sensitive N170 Response Is Enhanced for Nude Bodies

**DOI:** 10.1371/journal.pone.0024408

**Published:** 2011-11-16

**Authors:** Jari K. Hietanen, Lauri Nummenmaa

**Affiliations:** 1 Human Information Processing Laboratory, School of Social Sciences and Humanities, University of Tampere, Tampere, Finland; 2 Brain Research Unit, Low Temperature Laboratory, Aalto University School of Science and Technology, Espoo, Finland; 3 Department of Biomedical Engineering and Computational Science, Aalto University School of Science and Technology, Espoo, Finland; 4 Turku PET Centre, University of Turku, Turku, Finland; University of Leuven, Belgium

## Abstract

Recent event-related potential studies have shown that the occipitotemporal N170 component - best known for its sensitivity to faces - is also sensitive to perception of human bodies. Considering that in the timescale of evolution clothing is a relatively new invention that hides the bodily features relevant for sexual selection and arousal, we investigated whether the early N170 brain response would be enhanced to nude over clothed bodies. In two experiments, we measured N170 responses to nude bodies, bodies wearing swimsuits, clothed bodies, faces, and control stimuli (cars). We found that the N170 amplitude was larger to opposite and same-sex nude vs. clothed bodies. Moreover, the N170 amplitude increased linearly as the amount of clothing decreased from full clothing via swimsuits to nude bodies. Strikingly, the N170 response to nude bodies was even greater than that to faces, and the N170 amplitude to bodies was independent of whether the face of the bodies was visible or not. All human stimuli evoked greater N170 responses than did the control stimulus. Autonomic measurements and self-evaluations showed that nude bodies were affectively more arousing compared to the other stimulus categories. We conclude that the early visual processing of human bodies is sensitive to the visibility of the sex-related features of human bodies and that the visual processing of other people's nude bodies is enhanced in the brain. This enhancement is likely to reflect affective arousal elicited by nude bodies. Such facilitated visual processing of other people's nude bodies is possibly beneficial in identifying potential mating partners and competitors, and for triggering sexual behavior.

## Introduction

Without any doubt, other human beings are the most important visual objects in our environment. Compatible with this, cognitive neuroscience has revealed that the perception of other human beings is based on brain mechanisms specifically devoted to processing visual information from this socially and biologically relevant class of stimuli [1]. Much research has focused on neurocognitive mechanisms subserving perception of human faces and bodies as they both provide information necessary for social interaction and interpersonal relationships.

Several lines of evidence suggest that both human and sub-human primate brains contain cells specialized in processing of facial information [2]–[4]. For example, functional neuroimaging studies have identified an interconnected occipitotemporal neural network which shows face-selective response properties and high level of specialization in processing of the facial image [3], [5], [6]. Perception of human bodies is also subserved by a specialized brain network [7]–[9]. Paralleling the functional properties of the face-sensitive mechanisms, these lateral and ventral occipito-temporal circuits are engaged more vigorously during the perception of human bodies and body parts than during the perception of inanimate objects, isolated body parts or faces [7], [10], [11].

Electroencephalography (EEG) and magnetoencephalography (MEG) studies have investigated the early stages of visual processing of human faces and bodies. These studies have identified an event-related potential (ERP) and its magnetic counterpart recorded over occipito-temporal regions peaking between 140–200 ms after stimulus onset and being more sensitive to faces than to other objects [12]–[15]. Because of the typical peak latency (170 ms) of this negative potential, it is often referred to as N170 response. However, whether the N170 reflects only processes related to face perception is still a matter of great debate [14], [16], [17]. It has indeed been shown that the perception of bodies also triggers a profound N170 component whose amplitude is greater than that triggered by objects, but smaller than [18], [19] or similar [20], [21] to that triggered by faces. The body-sensitivity of the N170 response could be explained by arguing that the N170 response reflects the processing of configurally represented information [22] which is important both for face [23] and body [24] perception. Compatible with this view, the N170 response to both faces and bodies shows response characteristics considered typical for configural or holistic processing of visual stimuli, such as sensitivity to spatial inversion of stimuli [19], [25]–[27]. However, considering that the potential distribution and source localization of the N170 response differ between bodies and faces [18], [20], it is reasonable to assume that the N170 response does not reflect mere general configural processing of visual objects. More likely, it is driven by activation of the above-mentioned occipito-temporal areas specialized for processing of faces and bodies.

In all the aforementioned electrophysiological studies investigating the early visual processing of body information [18]–[21], [26], [27], the bodies were presented as wearing clothes. However, during the evolution, the cortical networks specialized in body perception have probably been tuned to respond to nude bodies. Although clothing provides cues to gender, sexual status, and rank in many cultures, clothing is typically used to restrict the visibility of the body, especially the primary and secondary sexual cues, and thus clothing hides bodily features relevant for sexual selection and arousal. Among primates, identification of mating partners relies extensively on the visual system [28] and humans, like other primates, display highly selective preferences in viewing the sexual signals of conspecifics [29], [30]. Perception of these signals and their evaluation as positive leads to physiological arousal, which can subsequently trigger sexual behaviors and ultimately copulation [31]. Efficient perception of sexual signals and categorization of conspecifics as potential mating partners or competitors is thus essential for both sexual selection and ensuring reproduction in primates.

Earliest recorded signs of clothing date to 36,000 BCE [32], although genetic and molecular clock estimates of the head and body lice – the latter having little chance of surviving on naked human body – suggest that body lice have originated already 72,000±42,000 years ago, which could coincide with the beginning of frequent use of clothing [33]. Thus, considering that the use of clothing has a relatively short history in the time-scale of evolution, it is possible that the responses of the brain networks specialized in body perception could show attenuated responses towards bodies wearing clothing. It has actually been proposed that colour vision might have evolved in primates for discriminating the spectral modulations on the skin of conspecifics [34]. In line with this, human visual system has been found to be particularly sensitive to detecting desaturated reddish targets resembling human skin tones [35]. Against this background, an interesting question arises: does the human brain show enhanced visual processing of nude over clothed bodies, and especially, if so, can we find the enhanced processing already at the early stages of visual processing?

As noted, nude bodies, especially of the opposite sex, are affectively pleasant and highly arousing stimuli [36], [37]. There is an extensive literature on affective picture processing showing that stimulus valence and arousal, in general, modulate several ERP components (for a review, see [38]). In this line of research, the high arousing, affectively pleasant stimuli have often included pictures showing nude people. However, none of these studies has investigated specifically the early visual processing of nude bodies. Furthermore, most prior studies have analyzed middle (200–300 ms) or long latency (>300 ms) responses triggered by complex pleasant scenes with varying content. There are, however, also studies that have shown affective modulation on short latency ERP responses. The evidence regarding the affective modulation of the P1 component is inconclusive and, if anything, it suggests that P1 is enhanced to stimuli with unpleasant valence (see [38]). However, reliable affective modulation has been observed for later N1 responses for pleasant [39]–[41] and unpleasant stimuli [40]. There is also some evidence that nude bodies might trigger enhanced early occipitotemporal responses. A MEG study measuring responses specifically to same-sex and opposite-sex nudes reported two early components which were larger to nude bodies than to neutral, non-human objects [42]. The earlier component, with a mean latency of 126 ms, was larger to male and female nudes compared to neutral pictures, but only in male participants, whereas the second component, with a mean latency of 203 ms, was larger to nudes than to neutral pictures in both sexes. However, the responses were averaged over all occipital and temporal channels and, more importantly, responses to nude bodies were compared with those to objects rather than with those to clothed bodies or faces. Thus, these studies do not reveal how nudity influences early visual processing of human bodies. Functional imaging studies with coarser time-scale have also confirmed that occipitotemporal responses are amplified for erotic pictures involving couples as well as single nude bodies (for a review, see Table 1 in [43]). However, due to the limits of temporal resolution of fMRI, these studies have also been unable to characterize the processing in the early visual stages. In sum, although the visual processing of sexually explicit material has been extensively investigated, our knowledge of how clothing affects the early visual responses to human bodies remains elusive.

In the present ERP experiments we tested whether the amplitude of the well-known face and body selective N170 component is dependent on whether the bodies were nude or clothed. Because N170 is known specifically for its sensitivity to faces, we also wanted to compare responses to nude and clothed bodies vs. faces. In two experiments we measured ERPs elicited by faces, nude bodies, bodies wearing sexually revealing clothing (swimsuits), fully clothed bodies as well as inanimate control objects (automobiles). Our prediction was straightforward: if the brain prioritizes the visual processing of nude bodies, N170 amplitude should be larger to nude than to clothed human bodies.

## Experiment 1

**Figure 1:**
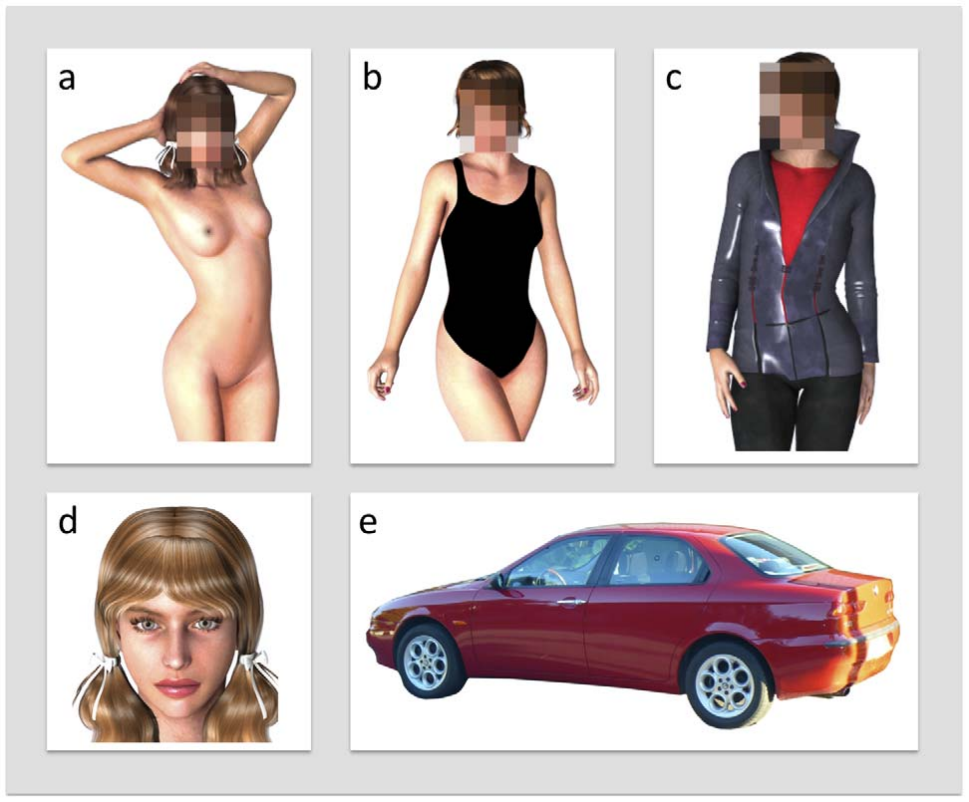
Illustration of the stimuli used in Experiments 1 and 2. In Experiment 1, pictures of nude bodies (a), bodies wearing swimsuits (b), faces (d), and cars (e) were used. In Experiment 2, pictures of fully clothed bodies (c) were presented instead of bodies wearing swimsuits. In Experiment 1, the heads of the bodies were masked by pixellation. In Experiment 2, the bodies were presented with both intact and masked heads, and the face and car stimuli were presented in intact and masked forms. In both experiments, the face and body pictures were color photographs of human models. The images shown here do not depict the actual stimuli but are intended only as examples.

In Experiment 1, color pictures of cars, faces, nude bodies, and bodies in swimsuits were presented as stimuli (see Fig. 1). In the pictures showing bodies, the head was masked. To match the nude and clothed bodies as well as possible, we used the same set of pictures for both conditions, except that we covered the nude bodies by drawing black swimsuits on top of them. This method also controlled for the possible effects of body posture on the responses. We were also interested in the possible effects of the models' sex on the responses to nude female and male bodies. For Experiment 1, we recruited only male participants because, in previous studies, the enhancement of brain activation in visual areas to erotic vs. neutral pictures has been shown to be larger among male than female participants [42], [44] and, moreover, male participants' physiological and evaluative responses have been found to be markedly discriminative to opposite-sex vs. same-sex sexual stimuli [42], [45]. In Experiment 1 (and also in Experiment 2), we restricted the data analysis to the N170 component due to its well-known face and body-sensitive response properties.

### Materials and Methods

#### Ethics Statement

The research was conducted according to the ethical standards of the American Psychological Association (APA). According to Finnish regulations (Act on Medical Research and Decree on Medical Research 1999, amended 2010), specific ethics approval was not necessary for this study. A written informed consent was obtained from all participants.

#### Participants

Fifteen healthy male volunteers with normal or corrected-to-normal vision participated in the experiment (age: *M* = 28.27; *SD* = 8.30; range 20–47 years). One of the participants was left-handed and all the others were right-handed.

#### Stimuli

Color pictures of faces, nude bodies, bodies wearing swimsuits, and cars were used as stimuli (see Fig. 1). The stimuli were downloaded from different websites. Face stimuli were generated by clipping the faces from the full-body pictures, hence the relative sizes of the face and body stimuli reflected the natural biological structure of the human face and body. For the swimsuit pictures, black swimsuits (one-piece swimsuits for females, square cut swimsuits for males) were drawn on top of the nude bodies with Adobe Photoshop 11 drawing tools. The models in the pictures showing bodies were standing in typical modeling postures. In the pictures showing bodies, a rectangular shaped area around the head was masked by means of decreasing the resolution in the head area to an average of 4 pixels per inch. In the stimuli showing humans, half included female models and the other half male models. The deviant target stimulus category (see below) included color pictures of various animals. There were 20 pictures in each category.

The stimulus size (horizontal×vertical) at the viewing distance of 78 cm was 6°×8° for faces (8.2 cm×10.9 cm), 7°×15° for bodies (9.5 cm×20.5 cm), 18°×12° for cars (24.7 cm×16.3 cm), and 13°×12° (17.2 cm×16.4 cm) for target animals. All stimuli were cut out from the backdrop, and shown against a white background. The fixation point was a black plus (0.7°×0.7°; 1 cm×1 cm). The stimuli were shown on a 17″ LCD-monitor set to resolution 1024×768 and with a refresh rate of 75 Hz. Neuroscan Stim software controller stimulus presentation.

#### Procedure

Participants were seated comfortably in an armchair in front of a monitor. The laboratory room was dimly lit. In the ERP measurements, the presentation of the stimulus pictures was divided into four blocks of equal length. The stimuli were presented in random order with the restriction that two animal pictures (targets) were not shown in succession. Pictures were shown for 500 ms with an inter-trial interval (ITI) of 1500 ms. A fixation point was shown during ITI in the middle of the screen. Before the actual experiment the participants practiced the task with a short training block (with stimulus examples not included in the experimental blocks). Participants sat in a chair holding a response button in their right hand. They were instructed to press the button whenever they saw an animal. They were asked to fixate at the fixation point during the stimulus set. There was a short pause between the four blocks.

#### EEG recording and statistical data analysis

Continuous EEG was recorded from the scalp with 21 electrodes (Ag/AgCl) mounted on an elastic cap (Electro-Cap International, Inc.). A 10–20 electrode positioning system was used. Online, the signal was referenced to nosetip. Mild skin abrasion was used to reduce the electrode impedances below 5 kΩ. Vertical (VEOG) and horizontal (HEOG) electro-oculogram was recorded with bipolar channels from sites above and below the midpoint of the left eye and beside the outer canthi of each eye. The EEG was band-pass filtered from 0.05 to 100 Hz and amplified with a gain of 5000 before storing on a computer disk at a sample rate of 1000 Hz (Neuroscan/Synamps).

Off-line, the EEG signal was digitally filtered using a 30 Hz lowpass filter and re-referenced to an average of all recording channels. The data were segmented to 600 ms epochs starting 100 ms prior to stimulus presentation. The segments were baseline-corrected against the mean voltage during the 100-ms pre-stimulus period. Segments with eye movements and blinks were excluded from further analyses using ±100 µV thresholds for the HEOG and VEOG. Average waveforms for each participant in each condition were calculated from the accepted trials. For N170, peak amplitude and its latency were analyzed within a 110–180-ms post-stimulus time window. The amplitude and latency analyses were based on ERPs recorded from electrodes T5 and T6. The data were normally distributed and thus analyzed with analyses of variance.

Greenhouse-Geisser correction procedure was applied to analysis of variance when necessary. For the sake of brevity, the original degrees of freedom are reported. For all multiple comparisons, Šidák correction was performed.

### Results

#### N170 amplitude

**Figure 2:**
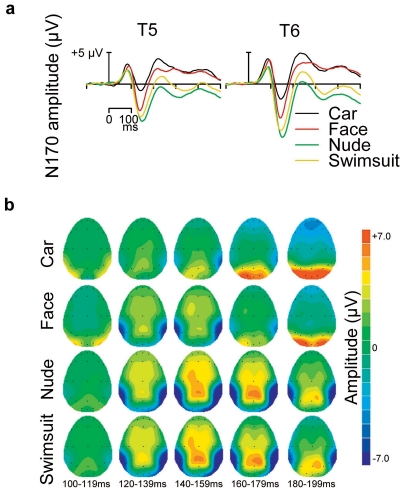
Brain responses to different stimuli in Experiment 1. (a) ERPs from occipitotemporal channels T5 (left) and T6 (right) to cars, faces, nude bodies, and bodies in swimsuits. The responses are averaged across stimulus sex. (b) Scalp topographies of the mean voltage amplitudes for different stimulus types in five consecutive 20-ms time windows starting at 100 ms post-stimulus.

**Figure 3:**
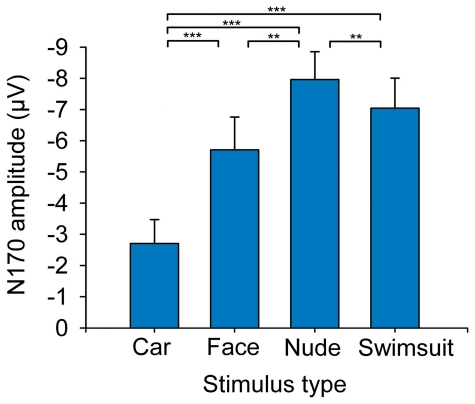
Histograms (mean + s.e.m.) showing the amplitude of the N170 response to different stimuli in Experiment 1. Amplitudes are averaged across recording channel and stimulus sex. (**P<.01; ***P<.001).

Figure 2 shows ERPs from two occipito-temporal (T5, T6) channels and scalp topographies of the mean voltage amplitudes for different stimulus types (averaged across stimulus sex). All stimulus categories elicited a prominent N170 response on these channels. For faces and bodies, the amplitude of the N170 response was at its largest on these channels (see Supporting Information, Fig. S1). A Stimulus (car, face, nude body, swimsuit body)×Laterality (left vs. right) ANOVA on the N170 amplitude showed a main effect of stimulus type (*F*(3,42) = 34.8, *P*<.001). Pairwise comparisons indicated that the N170 response to all human stimuli was greater than the response to cars (all *P*s<.001) (Fig. 3). More interestingly, the N170 amplitude to nude bodies (*M* = −8.0 µV) was greater than the response to swimsuit bodies (*M* = −7.0 µV) or faces (*M* = −5.7 µV) (both *P*s<.01). The difference between swimsuit bodies and faces was not significant (*P* = .108). The voltage topographies suggest that perception of nude bodies resulted in longer-lasting, more extensive, and more posteriorly extending negativity than perception of other stimulus classes (see also Supporting Information, Fig. S2).

Next we tested whether the N170 responses to human stimuli were affected by the sex of the model. A Stimulus (face, nude body, swimsuit)×Stimulus Sex (male vs. female)×Laterality ANOVA showed the effects of Stimulus (*F*(2,28) = 13.0, *P*<.001), and Stimulus×Stimulus Sex interaction (*F*(2,28) = 6.7, *P* = .013). Pairwise comparisons indicated that the N170 amplitude was greater to naked females than males (*M* = −8.2 µV vs. −7.7 µV, *P* = .034), whereas there was no difference in response to female and male faces (*M* = −5.8 µV vs. −5.6 µV, *P* = .316). The N170 amplitude was lower for female than male swimsuit bodies (*M* = −6.6 µV vs. −7.5 µV, *P* = .049).

#### N170 latency

The N170 latency data showed the main effects of stimulus (*F*(3,42) = 10.7, *P*<.001), and laterality (*F*(1,14) = 5.8, *P* = .03). Pairwise comparisons indicated that the N170 latency was longer to nude bodies (*M* = 153 ms) than to swimsuit bodies (*M* = 146 ms), faces (*M* = 141 ms), and cars (*M* = 141 ms) (*P* = .02). The N170 latency was longer in the right (*M* = 147 ms) than left (*M* = 143 ms) hemisphere. The N170 latencies to human stimuli were not affected by the sex of the model.

### Discussion

Our main finding was that clothing modulated the N170 response to bodies: N170 amplitude was larger to nude bodies than to the bodies wearing swimsuits, while all body stimuli elicited larger N170 responses than control objects (cars). In general, these results are compatible with the prior studies on body-sensitivity of the N170 component [18]–[21], [26], [27] as well as the extensive line of research showing enhanced ERP responses to affectively arousing stimuli [38]. Specifically, our findings converge with the experiments showing enhanced N1 responses to affective stimuli [39]–[41]. The present experiment nevertheless goes beyond the previous findings in two important aspects. First, we directly compared visual responses to single human bodies which were either nude or covered (by a swimsuit) and, second, we showed the effect of nudity on N170 response measured from those occipito-temporal channels showing typically high sensitivity for human faces and bodies. Our results thus confirm that nudity of human bodies is detected early on during visual processing, and that the human brain exhibits enhanced visual processing to other people's nude bodies. Interestingly, the N170 response to nude bodies was *even greater* than that to faces, even though the N170 response to faces was at its largest on the chosen T5/T6 channels. The amplitude difference between swimsuit bodies and faces was not statistically significant. Given the sheer number of studies showing that faces elicit the most pronounced N170 amplitudes [12]–[14], our findings showing the largest N170 amplitudes to nude bodies is somewhat surprising.

Experiment 1 involved only male participants, and their N170 amplitude was greater to nude female bodies than to nude male bodies. This fits with prior findings showing that male participant's evaluative and looking-time responses [42], [45], [46] are different for same vs. opposite-sex nude bodies. We extended these findings to early visual cortical responses to body stimuli. On the contrary, stimulus sex had no effect on N170 to faces, which is compatible with previous results showing no difference in N170 responses to same-sex vs. opposite-sex faces [47].

## Experiment 2

Experiment 2 had three major aims. First, we investigated whether our findings on the enhanced N170 component to nude vs. clothed and opposite vs. same-sex nude bodies would also be generalized to female participants. To that end, we measured ERPs from both male and female subjects. To make our results on the body-selective N170 response directly comparable with prior studies, we presented participants with nude and fully clothed male and female stimuli. Second, considering the importance of facial information for sexual behavior (e.g. [48]), we tested whether the N170 amplitude would be even more pronounced to nude bodies with visible heads/faces than to nude bodies with masked heads. This also allowed us to study the effects of face visibility on N170 responses to human bodies in general. To have a full factorial design in our experiment, we presented all our stimulus categories both in intact and masked versions. In the masked nude and clothed body pictures only the heads were pixellated (see sample stimuli in Fig. 1). Finally, we evaluated whether the enhanced N170 response to nude bodies would be associated with elevated physiological arousal and self-reported affective experiences. To this end we measured skin conductance responses (SCR) and subjective evaluations of arousal and emotional valence occasioned by the stimuli, and correlated these with the corresponding N170 amplitudes.

### Materials and Methods

#### Ethics Statement

The research was conducted according to the ethical standards of the American Psychological Association (APA). According to Finnish regulations (Act on Medical Research and Decree on Medical Research 1999, amended 2010), specific ethics approval was not necessary for this study. A written informed consent was obtained from all participants.

#### Participants

Thirty-two (16 females) healthy volunteers with normal or corrected-to-normal vision participated in the experiment (age M = 24.47, SD = 8.32, range 19–66 years). One of the males was left-handed while all the other participants were right-handed. The EEG data of one male participant were not recorded because of technical problems.

#### Stimuli

Color pictures of faces, nude bodies, clothed bodies, and cars were used as stimuli (see illustrations in Fig. 1). The models in both the nude and clothed body pictures were standing in typical modeling postures. Half of the human pictures involved female models and the other half male models. Like in Experiment 1, the deviant target stimulus category included color pictures of various animals. The size of the stimuli and their presentation conditions were similar to those in Experiment 1. Different stimulus sets were used for the ERP and SCR experiments. The stimuli for the ERP experiment were 20 pictures of faces (male/female), nude bodies (male/female), clothed bodies (male/female), and cars. There were two versions of each picture: intact and masked. In the masked face and car pictures, the head/car was rendered unrecognizable by means of pixellation (*4* pixels per inch), whereas for masked nude and clothed body pictures only the heads were pixellated. The target stimulus category included 20 pictures of different animals. The stimuli for the SCR measurements consisted of color pictures of faces, naked bodies with masked face, clothed bodies with masked face, and cars. There were thus six categories of human pictures (males and females) and pictures of cars. Five different pictures from each category were presented. Pictures of animals served as targets.

#### Procedure

Participants were seated comfortably in an armchair in front of a monitor. The laboratory room was dimly lit. The experimental session began with the SCR measurements where each of the 38 pictures (including 3 pictures of animal targets) was presented for 3 seconds with an ITI of 15 seconds. During the ITI, a fixation point was shown at the center of the screen. The stimuli were presented in pseudo-random order - one picture from each category was shown before the next presentation from this category - within two blocks. Participants were instructed to sit still and comfortably while looking at the pictures and to press the left mouse button every time they saw a picture of an animal. The procedure for the ERP measurements was similar to that in Experiment 1.

Immediately after the SCR and ERP measurements, participants rated their experiences of valence and arousal while viewing the face, body and car stimuli using the SAM (Self-Assessment Manikin [49]). After this the electrodes were removed and the participants were allowed to clean themselves. Finally, participants completed The Sell Assessment of Sexual Orientation –questionnaire [50]. This questionnaire measures sexual attraction towards males and females, amount of sexual activity with males and females, and sexual identity (homo/heterosexuality). For assessing our participants' sexual orientation, we analyzed answers to an item where the participants were asked to rate their degree of homosexuality on a 7-point scale (1 = not at all homosexual; 7 = extremely homosexual).

#### EEG and SCR recording and statistical data analysis

The recording and analysis of the EEG/ERP data were similar to those in Experiment 1. Skin conductance was measured with Ag/AgCl electrodes (diameter 8 mm). The palmar surfaces on the medial phalanxes of the index and middle fingers of the left hand were cleaned and the electrodes filled with paste were attached to the corresponding locations. The signal was acquired with a GSR amplifier supplying constant-voltage AC excitation (22 mV) (ADInstruments). Recordings were done with Power Lab 400 equipment, using Power Lab Chart 3.6 program running on a Power Macintosh 7100/80 computer. The sampling rate was 100 Hz. The skin conductance response (SCR) was defined as the maximum change from the baseline level (at the stimulus onset) during a 4-second time period starting 1 second after stimulus onset. The data were averaged in each condition for each participant. Responses contaminated, for example, by participant's body movements or by technical problems were eliminated from the analysis. Of all the trials 28.3% were discarded on the basis of these criteria. To normalize the data for the statistical analyses, a log transformation [log (SCR+1)] was performed.

Greenhouse-Geisser correction procedure was applied to analysis of variance when necessary. For the sake of brevity, the original degrees of freedom are reported. For all multiple comparisons, Šidák correction was performed.

### Results

#### N170 amplitude

**Figure 4:**
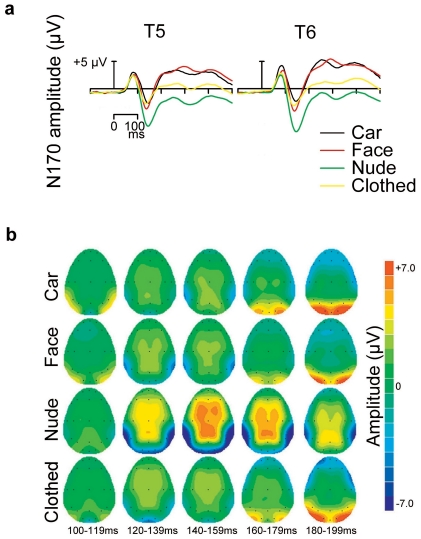
Brain responses to different stimuli in Experiment 2. (a) ERPs from occipitotemporal channels T5 (left) and T6 (right) to cars, faces, nude bodies, and clothed bodies. The responses are averaged across stimulus and participant's sex. (b) Scalp topographies of the mean voltage amplitudes for different stimulus types in five consecutive 20-ms time windows starting at 100 ms post-stimulus. For nude and clothed bodies, the data are from the trials with masked head.

**Figure 5:**
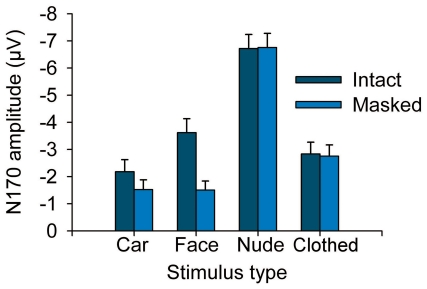
Histograms (mean + s.e.m.) showing the amplitude of the N170 responses in Experiment 2. Amplitudes are shown to intact and masked versions of different stimuli, averaged across the recording channel, stimulus sex, and participant's sex.

Again, all stimulus categories elicited a prominent N170 response (Fig. 4a). For faces and bodies, the amplitude of the N170 response was at its largest on these channels (see Supporting Information, Fig. S3). The analysis of the N170 amplitude data showed a significant effect of stimulus (*F*(3,90) = 140.51, *P*<.001). N170 was significantly greater to nude bodies (*M* = −6.7 µV) than to all other stimulus categories (all *P*s<.001). N170 amplitude to faces (*M* = −2.6 µV) and clothed bodies (*M* = −2.8 µV) was also greater than that to cars (*M* = −1.9 µV, both *P*s<.01), but amplitudes for faces and bodies did not differ (*P* = .825). In addition, Stimulus×Masking (*F*(3,90) = 20.00, *P*<.001) and Laterality×Stimulus (*F*(3,90) = 3.90, *P* = .026) interactions were significant. The masking decreased the N170 amplitude to cars (intact vs. masked; *M* = −2.2 µV vs. −1.5 µV), and faces (*M* = −3.6 µV vs. −1.5 µV), but had no effect on nude (*M* = −6.7 µV vs. −6.8 µV) and clothed (*M* = −2.8 µV vs. −2.8 µV) bodies (Fig. 5). Additionally, the N170 amplitude was greater in the right than left hemisphere for faces and bodies, whereas the opposite was true for cars. Like in Experiment 1, the scalp topographies showed again a long-lasting and extensive negativity for nude bodies (see Fig. 4b and Supporting Information, Fig. S4).

We also analyzed the effects of stimulus and participant sex on the N170 amplitudes to human stimuli. In order to make direct comparisons with the results from Experiment 1, the analyses were restricted to body pictures with masked heads. A Stimulus (face, nude body, clothed)×Stimulus Sex×Laterality×Participant Sex ANOVA showed, apart from the main effect of stimulus, the main effect of Stimulus Sex (*F*(1, 29) = 4.67, *P* = .039). N170 amplitude was larger to female (*M* = −4.5 µV) than male (*M* = −4.3 µV), stimuli. However, the effect of stimulus sex was qualified by a borderline Stimulus Sex×Participant Sex interaction (*F*(1, 29) = 3.77, *P* = .062). As we had clear *a priori* predictions about this interaction, planned contrasts were conducted. As in Experiment 1, males showed larger N170 responses to female (*M* = −5.3 µV) than to male (*M* = −4.8 µV, *P*<.01) pictures. On the contrary, stimulus sex had no effect on N170 amplitudes in female subjects (male vs. female pictures, *M* = −3.8 µV vs. −3.8 µV, *P* = .891).

We also analyzed whether the effects of stimulus and participant sex were affected by the participants' sexual orientation. Among our 31 participants contributing to the ERP results, 21 (11 males, 10 females) considered themselves as ‘not at all’ homosexual. The rest described themselves as slightly homosexual (4 males, 3 females), mildly homosexual (1 female), or moderately homosexual (2 females). When the effects of stimulus and participant sex on the N170 amplitudes to human stimuli were analyzed for the ‘not at all homosexual’ participants, the results remained exactly the same as above. For males, N170 was larger to all female vs. male stimuli (*P* = .002), whereas there was no effect of stimulus sex for females (*P*>.5). Although the number of participants considering themselves as at least ‘slightly homosexual’ was very low, we nevertheless analyzed the data for these participants also. A similar ANOVA as above showed, besides the effect of stimulus, a Stimulus×Stimulus Sex×Participant Sex interaction (*F*(2, 16) = 5.12, *P* = .019). For these male participants (n = 4), the effect of stimulus sex was not significant (male vs. female pictures, *M* = −4.9 µV vs. −4.9 µV, *P* = .841). Instead, for the females (n = 6), a Stimulus×Stimulus Sex interaction approached significance (*F*(2, 10) = 3.68, *P* = .063). Pairwise comparisons indicated that the N170 amplitude was marginally greater in reaction to naked females than males (*M* = −7.5 µV vs. −6.3 µV, *P* = .076), whereas there was no difference in response to female and male faces (*M* = −3.4 µV vs. −3.8 µV) or to female and male clothed bodies (*M* = −2.8 µV vs. −2.7 µV).

**Figure 6:**
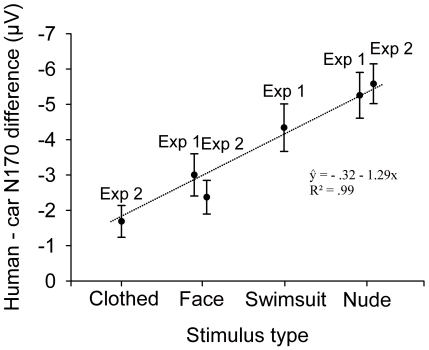
The N170 amplitude modulation as a function of the degree of clothing. The graph illustrates the N170 amplitude difference (mean + s.e.m.) calculated between each human stimulus category and cars in Experiments 1 and 2. In Experiment 2, data from male participants only (n = 15) were included. The regression line is based on the mean amplitude differences (for faces and nude bodies, averaged across data from Experiments 1 and 2).

Finally, we investigated the effect of the degree of clothing on N170 amplitude by pooling data from Experiments 1 and 2. As Experiment 1 only involved male participants, this analysis was confined to male participants only (additional analyses confirmed that the following pattern of results remained the same had we included female participants from Experiment 2). Because the data on swimsuit and fully clothed bodies were collected from different experiments, we standardized the data from the human stimulus conditions in Experiments 1 and 2 by computing amplitude differences between cars and each other stimulus condition in the respective experiment. This analysis showed that the N170 amplitude increased linearly (R^2^ for Least Squares regression line = .99) as the degree of clothing decreased (Fig. 6). The amplitude differences between responses to faces and nude bodies vs. cars were equal between Experiments 1 and 2 (faces: 3.0 µV vs. 2.4 µV; nude bodies: 5.3 µV vs. 5.6 µV; both *P*s>.4). This result provides further justification for comparing the responses to swimsuit vs. fully clothed bodies despite the data were collected from separate experiments.

#### N170 latency

The analysis on the N170 latency data showed a main effects of stimulus (*F*(3,90) = 10.17, *P*<.001) and masking (*F*(1,30) = 18.62, *P*<.001), as well as Stimulus×Masking interaction (*F*(3,90) = 9.18, *P*<.001). Overall, the N170 latency was significantly longer to cars (*M* = 151 ms) and nude bodies (*M* = 150 ms) compared to clothed bodies (*M* = 142 ms, both *P*s<.001). The N170 latency to faces did not differ from the other stimulus categories *M* = 147 ms). The masking had a greater influence on N170 latency to cars (intact vs. masked; *M_intact_ = *148 ms vs. *M_masked_* 154 ms), and faces (*M* = 140 ms vs. 154 ms) than on N170 latency to nude (*M* = 148 vs. 152 ms) and clothed (*M* = 143 vs. 142 ms) bodies.

The latency analysis involving the effects of stimulus and participant sex on the N170 latencies to human stimuli showed the main effect of stimulus, as expected, and also the main effect of participant sex (*F*(1,29) = 7.62, *P*<.01). Overall, the N170 latency was significantly longer to male (*M* = 150 ms) than female (*M* = 140 ms) participants.

#### SCR and subjective arousal and valence evaluations

**Figure 7:**
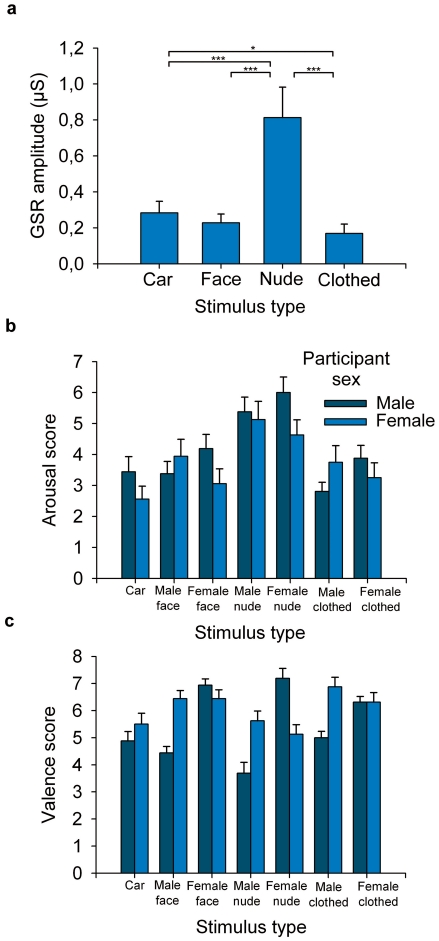
Arousal and valence responses. (a) Skin conductance responses (mean + s.e.m.) to pictures of faces, nude bodies with masked heads, clothed bodies with masked heads, and cars. (*P<.05; ***P<.001). (b) Subjective arousal ratings (mean + s.e.m.) to different stimulus categories. (c) Subjective valence ratings (mean + s.e.m.) to different stimulus categories.

Skin conductance responses (Fig. 7a) were first analyzed by one-way repeated measures ANOVA comparing faces vs. nude bodies with masked heads vs. clothed bodies with masked heads vs. cars. The effect of stimulus type was significant (*F*(3,93) = 22.08, *P*<.001). SCR to nude bodies was greater (*M* = 0.813 µMho) than SCR to dressed bodies (*M* = 0.169 µMho), faces (*M* = 0.228 µMho), and cars (*M* = 0.284 µMho) (all *P*s<.001). SCR to cars was also greater than SCR to clothed bodies (*P* = .012). A follow-up analysis for the human stimuli (Stimulus×Stimulus Sex×Participant Sex ANOVA) showed that the SCRs to male and female human stimuli were not affected by the participant's gender. We also tested whether the physiological arousal triggered by the stimuli would be associated with N170 amplitudes. To that end, we took the overall means of SCRs and N170 amplitudes to each stimulus category and computed their rank order correlation (Spearman's rho). However, the correlation failed to reach significance (*r_s_* = −.4, *P* = .6).

Compatible with the autonomic responses, the effect of stimulus type was also significant for the subjective arousal ratings (*F*(3,93) = 30.59, *P*<.001) (Fig. 7b). Arousal ratings were higher to nude bodies (*M* = 5.3) than to all other categories (all *P*s<.001). Ratings to faces (*M* = 3.6), clothed bodies (*M* = 3.4), or cars (*M* = 3.0) did not differ from each other. Similarly to SCRs, we computed the rank correlation between the mean self-reported arousal ratings and N170 amplitudes to each stimulus category. This revealed a strong association between the self-reported arousal scores and N170 amplitudes (*r_s_* = −1.0, *P*<.001). Analysis of the human stimuli showed that the interaction between stimulus sex and participant sex (*F*(1,30) = 9.46, *P*<.01) was significant. For males, the arousal ratings were higher for female (*M* = 4.7) than male (*M* = 3.9) models, whereas for female participants the arousal ratings were higher for male (*M* = 4.3) than female (*M* = 3.6) models.

The effect of stimulus type (cars, faces, nude bodies, clothed bodies; averaged across stimulus gender and subject gender) on valence ratings was significant (*F*(3, 93) = 6.32, *P*<.01) (Fig. 7c). Valence ratings to faces (*M* = 6.1) and to clothed bodies (*M* = 6.1) were higher than those to nude bodies (*M* = 5.4) or cars (*M* = 5.21) (all *P*s<.05). There were no other differences. The mean self-reported valence ratings and mean N170 amplitudes to each stimulus category were not correlated (*r_s_* = −.2, *P* = .8). A Stimulus×Stimulus Sex×Participant Sex ANOVA on valence ratings to human stimuli showed that all main effects as well as the interactions were statistically significant (all *P*s<.05). These effects reflected the fact that for male participants, the valence ratings were higher for all female than male stimuli (*M* = 6.8 vs. 4.4, averaged across stimulus categories), whereas, for females, the opposite-sex preference was smaller (*M* = 6.4 vs. 6.0, averaged across stimulus categories) and, moreover, the female participants rated male and female faces equally pleasant (both *M*s = 6.5).

### Discussion

In Experiment 2 we replicated our findings on larger N170 amplitudes to nude vs. clothed bodies and confirmed that this occurs for both male and female participants. Moreover, combined analysis of Experiments 1 and 2 demonstrated that N170 to bodies was sensitive to the degree of clothing: the N170 amplitude increased linearly as the amount of clothing decreased from full clothing via swimsuits to nude bodies. These results show that early visual processing of human bodies is sensitive to the visibility of the sex-related features of human bodies and that visual processing of other people's nude bodies is enhanced in the brain.

In Experiments 2 male subjects exhibited larger the N170 responses to all opposite-sex compared to same-sex human stimuli, whereas in Experiment 1 this bias towards opposite-sex stimuli was restricted to nude bodies only. For female participants, the stimulus sex had no effect on N170 responses. These findings are compatible with the prevailing view of sexual responsiveness suggesting greater discrimination of physiological responses to sexually arousing opposite-sex vs. same-sex stimuli among males than females [42], [45], [46], [51], [52]. Comparison of the results between those participants who considered themselves as ‘not at all’ homosexual and those who considered themselves as at least slightly homosexually oriented suggested that the participant's sexual orientation might be modulating the N170 responses to nude bodies. Previous electrophysiological studies have shown a late, attention-related ERP component, Contingent Negative Variation (CNV), to be larger to female than male nudes in heterosexual but not in homosexual males [53]. Recently, sexual orientation has also been shown to influence on attention orienting to visually suppressed, nude male and female bodies [54]. In future studies it will be interesting to investigate the effect of stimulus sex on N170 response to nude bodies in homosexually oriented male and female participants. If the results confirmed that the discrimination between responses to female vs. male bodies is different in heterosexually and homosexually oriented participants, the results would strengthen the view that the visual system enhances the early processing of information relevant to one's sexual behavior.

One of the most surprising findings in Experiment 1 was that the N170 amplitude to nude bodies was larger than that to faces. We successfully replicated this effect in Experiment 2, and demonstrated that the N170 response to bodies was strongly modulated by nudity in both male and female participants. In our stimulus sets, the face images were somewhat smaller in size compared to the body images. To keep the relative size of the bodies and faces similar to that under natural conditions, the heads were clipped away from the full-body stimuli without doing any rescaling of the stimuli. This obviously raises the question about whether the larger size of the body versus face images could explain the larger N170 amplitude to nude bodies vs. faces. This is nevertheless unlikely for three reasons. First, the N170 amplitude differentiated between the nude and swimsuit bodies as well as nude and clothed bodies even though their size was exactly the same. Second, the order of physical stimulus size (from the smallest to the largest) was faces, bodies, and cars, whereas the order of the N170 amplitude size was cars, faces, and nude bodies. Third, the N170 (N1) amplitude has been shown to be unaffected by the size of the stimulus [55].

The results of Experiment 2 also showed that visibility of faces had no effect on the N170 amplitude evoked by the nude or clothed bodies. Now, one could argue that the N170 responses to bodies were not reflecting only body-related visual processing, but that face-related visual processing could also have contributed to the responses. Namely, a recent fMRI study has shown that presenting a body in the context of a masked head elicits enhanced activity in the face-sensitive temporal brain regions (fusiform face area, FFA) compared to, for example, presentation of the masked head alone [56]. One could thus argue that maybe our N170 responses to bodies would have reflected summed responses to bodies *and* faces, the latter being triggered by the body context. However, considering that Experiment 2 showed that i) N170 amplitudes were larger for both masked and intact nude versus clothed bodies and ii) N170 amplitudes to bodies with masked and intact faces were equal, we do not find this possibility likely. One previous study (ref. [27] Fig. 5), found *larger* N170 response to human bodies with absent heads compared to those with heads, whereas our results, as noted above, indicated no effect of the presence of a intact vs. masked head. It is possible that removal of the head altogether (cf. [27]), results in rather an unusual picture of a “decapitated” human body, and this kind of a stimulus may evoke affective responses leading to enhanced N170 responses. In the present experiment, the head was masked by image pixellation rather than removed altogether. Our results show that even though facial information is important for human sexual behavior (e.g. [48]), the N170 response to nude bodies was not affected by the visibility of the face. In other words, facial information did not contribute to the enhancement of early visual processing to nude human bodies, but resulted from detection and processing of features confined to the body area. Accordingly, it is likely that the facial information related to sexual arousal is processed at a later stage, and is thus not reflected in the early face- and body-selective ERP components.

The measurements of autonomic responses and self-ratings of arousal showed higher arousal to nude bodies than to other stimulus categories. Furthermore, the mean self-reported arousal to different stimulus categories was significantly correlated with the mean N170 amplitudes. On the contrary, the valence ratings showed that the nude bodies were evaluated as less pleasant than the dressed bodies and faces, and the mean valence ratings did not correlate with the mean N170 amplitudes. These findings support the notion that the N170 enhancement to nude bodies is mediated by elevated affective arousal, and confirm that arousal - rather than affective valence – is responsible for the enhanced N170 amplitudes to nude bodies.

## Discussion

Our ERP data show for the first time that early visual processing of nude over clothed bodies is enhanced. Previous ERP studies had shown that the N170 component is sensitive to perception of human bodies [18]–[21], [26], [27]. The results from the present two experiments showed that the visual processing of male and female human bodies - as reflected by the occipito-temporal N170 response - was enhanced if their sex-related features were visible. Moreover, the results demonstrated that N170 to bodies was sensitive to the degree of sex-related features visible in the bodies: the N170 amplitude increased linearly as the amount of clothing decreased from full clothing via swimsuits to nude bodies. Importantly, our results also showed that the N170 response traditionally assumed to be most pronounced to human faces [12]–[14] turned out to be *even greater* to nude bodies than to faces. Altogether these results show that early face and body sensitive cortical responses are sensitive to the visibility of the sex-related features of human bodies and that the human brain exhibits enhanced visual processing of other people's nude bodies, a mechanism possibly beneficial in identifying potential mating partners and competitors.

But why would the N170 component be sensitive to the degree of nudity of the human bodies? Interpreting our findings against the dominant frameworks of the electrophysiology of face and body perception does not provide a satisfactory explanation. First, assuming that the N170 reflects the engagement of configural processing networks [22] one would have to argue that perception of nude human bodies increasingly activates the neural networks coding configurally or holistically represented visual information. However, this explanation is not very plausible, as our results showed that the nude bodies elicited an even greater N170 response than did human faces, the stimulus category whose perception is considered to be based on a particularly strong configural representation [23]. Second, another prominent hypothesis suggests that the N170 response reflects the functioning of the mechanisms sensitive to extensive experience with any category of visual stimuli (expertise hypothesis [17]). However, in this case, we would have to argue that our participants drawn from the volunteer pool of our university had had more exposure to nude bodies than to clothed bodies and faces. This is not likely, either.

A third and a more appealing explanation concerns the role of affective arousal in modulating the amplitude of the N170 response. Our measurements of autonomic responses and self-ratings support for this hypothesis: both measures showed higher arousal to nude bodies than to other stimulus categories, and the overall mean self-reported arousal scores to different stimulus categories were also correlated with the N170 amplitudes. This accords with the extensive literature showing enhanced ERP responses to affectively arousing stimuli (for a review, see [38]). A handful of previous studies has found affective modulation of early visual ERP responses (such as N1, e.g. [39]–[41]. We replicate these findings and show that particularly the face- and body-sensitive occipitotemporal N170 response shows significant sensitivity to a very specific affective signal, namely, the visibility of sex-related human body features.

In more general sense the present findings suggest that the face- and body-sensitive N170 response can be greatly modulated by the affective and motivational significance of the stimulus. Previous studies have already shown that the N170 amplitude is enhanced for emotionally arousing faces such as those with fearful rather than neutral facial expressions [57], [58], faces displaying gaze contact rather than gaze aversion (eye contact; [59], [60]), and familiar rather than unfamiliar faces [61]. Interestingly, in previous studies investigating the effects of expressive body postures on early visual processing, N170 has not been found to be modulated by body expressions [26], [62]. However, in one study the VPP (vertex positive potential), a positive counter-part of N170 recorded from the top of the head, was observed to be enhanced to fearful bodies [26]. One possible way to reconcile these seemingly discrepant results is that nude bodies are obviously much stronger and salient affective signals than, for example, fearful body postures. Accordingly, it is understandable that nude bodies but not bodily expressions trigger enhanced N170 amplitudes.

Taken together, our results suggest that the N170 component reflects two different types of processes: engagement of visual processing systems specialized for face and body perception, as well as affective-motivational processes tracking emotional arousal level. Configural versus featural processing of visual stimuli has typically been investigated by rotating the stimuli upside down. This manipulation disrupts configural coding and is known to interfere specifically with the perception of faces, bodies, and target objects of expertise. As inversion also increases the latency and sometimes also the amplitude of N170 to these stimuli [25], [26], [63], results from such studies convincingly suggest that N170 reflects visual processing of faces and bodies, and possibly also of other types of configurationally represented visual information. In line with this, we found larger N170 amplitudes for faces vs. cars in the absence of arousal differences between these categories. Accordingly, these differences must be due to an arousal-independent mechanism, such as differential engagement of the category-selective visual processing systems, or those subserving, for example, configural processing of visual information.

However, the present data highlight that inputs from brain regions encoding of salient and arousing visual stimuli also contribute significantly to the N170 wave. There is abundant evidence implicating the amygdala as one of the core regions involved in early encoding emotional features from sensory input [64], [65] including sexual cues [66]. It has been proposed [67], [68] that amygdala may accomplish some forms of affective evaluation even before visual information is transmitted to the occipitotemporal areas involved in object recognition. But recently it has been suggested that the cortex may have a more important role in processing of affective information than previously assumed. Pessoa and Adolphs [69] postulated a multiple-waves model emphasizing the existence of multiple parallel cortical routes and several ‘short-cut’ connections between lower and higher visual areas. Such connections would allow affective modulation of short-latency brain responses without a need to rely on subcortical processing.

Although we have emphasized the role of affective arousal in generating the enhanced N170 response to nude bodies, there may be other alternatives for explaining the present results. One possibility relates to attentional top-down effects on the N170 responses. Even though many studies have failed to find attention effects on the N170/M170 responses to faces [70]–[72], there are recent studies showing that the N170 response to faces can be sensitive to both spatial [73]–[75] and object-based [76], [77] selective attention. Thus, it is possible that more attention was allocated on nude vs. clothed bodies and the amplitude enhancement of the N170 response may have reflected this effect, at least to some extent. As the effects of affective arousal and attention are tightly intertwined, differentiating between these effects will be an important issue for future studies. Second, based on our results we can not strictly dissociate the contribution of body nudity per se and the contribution of affective arousal on enhanced N170 responses. Disentangling specifically the contribution of affective arousal would have required a control stimulus category not showing body nudity, but associated with positive valence and, importantly, eliciting high arousal comparable to that elicited by nude bodies. However, finding such a stimulus category is not straightforward. Another possibility would be replicating the present experiment with participants such as those with hypoactive sexual disorder, for whom nude bodies are not exceedingly arousing. These patients have, for example, been shown to experience lower level of sexual arousal during viewing of erotic films as compared to controls [78]. Finally, the N170 responses to a face have been shown to decrease when there is another face in the visual field, a finding interpreted to result from competition for recruiting a common population of neurons [79], [80]. Against this finding it is possible that the enhanced N170 response for naked vs. clothed bodies could reflect, in fact, attenuated N170 amplitudes to clothed bodies because of mutual suppressive competition among bodies and object shapes (clothes). In order to test this hypothesis, future studies could investigate, for example, whether presentation of mere clothes next to a nude body results in smaller N170 responses compared to those to the presentation of nude bodies only.

An important and yet open question is whether N170 elicited by faces vs. nude and clothed bodies reflects the activation of the same or different neural generators. In previous studies comparing the N170 response between clothed bodies and faces, some researchers have reported similar scalp topography for faces and bodies [26], whereas others have shown that the potential distribution and source localization differ between responses to bodies and faces [18], [20]. The N170 topography of the present study provided evidence for separate sources. However, in the present study the sensor density of 21 electrodes was too low to allow accurate source localization. This question must be addressed in future studies employing high-density electrode networks or magnetoencephalography. It will be of particular interest be to ascertain whether the processing of nude bodies engages only the same cortical sources as the processing of clothed bodies and faces, or whether the N170 response enhancement to nude bodies reflects activation of additional cortical-subcortical generators. Obviously, another great challenge for future research will be to reveal the visual features that make nude human bodies affectively arousing and subject to enhanced visual processing.

### Conclusions

We conclude that the human brain is tuned to detect sexual signals from human bodies rapidly, and that this categorization process is reflected in the face- and body-sensitive N170 component of the ERP wave. Such a perceptual ‘highway’ for processing of sexual cues is highly beneficial for triggering sexual behavior, and subsequently ensuring mating and reproduction. Furthermore, we argue that affective arousal can provide a significant contributory factor in generating the N170 response, providing further support for the models of rapid modulation of visual responses by emotional information [68].

## Supporting Information

Figure S1 N170 amplitudes measured from all posterior recording channels in Experiment 1. The largest amplitudes for stimuli from each category were measured as follows (averaged across left and right channels): car, T3/T4, *M* = −2.8 µV; face, T5/T6 *M* = −5.7 µV; nude, T5/T6 *M* = −8.0 µV; swimsuit, T5/T6, *M* = −7.0 µV.
(TIF)

Figure S2 Statistical comparisons for the scalp topographies of the mean voltage amplitudes between face vs. car, nude bodies vs. face, face vs. swimsuit bodies, and nude bodies vs. swimsuit bodies in Experiment 1. The statistical comparisons are plotted in five consecutive 20-ms time windows starting at 100 ms post-stimulus. The arrows indicate color codes corresponding to the critical t-values (*P*<.05).
(JPG)

Figure S3 N170 amplitudes to intact versions of the stimuli measured from all posterior recording channels in Experiment 2. The largest amplitudes for stimuli from each category were measured as follows (averaged across left and right channels): car, T3/T4, *M* = −2.6 µV; face, T5/T6 *M* = −3.6 µV; nude, T5/T6 *M* = −6.7 µV; clothed, O1/O2, *M* = −3.0 µV.
(TIF)

Figure S4 Statistical comparisons for the scalp topographies of the mean voltage amplitudes between face vs. car, nude bodies vs. face, face vs. clothed bodies, and nude bodies vs. clothed bodies in Experiment 2. The statistical comparisons are plotted in five consecutive 20-ms time windows starting at 100 ms post-stimulus. The arrows indicate color codes corresponding to the critical t-values (*P*<.05).
(JPG)
