# Supplementary figures and images for: The Naked Truth: The Face and Body Sensitive N170 Response Is Enhanced for Nude Bodies
Source: PLoS One. 2011 Nov 16;6(11):e24408. doi: 10.1371/journal.pone.0024408 (PMC3217929; doi:10.1371/journal.pone.0024408)

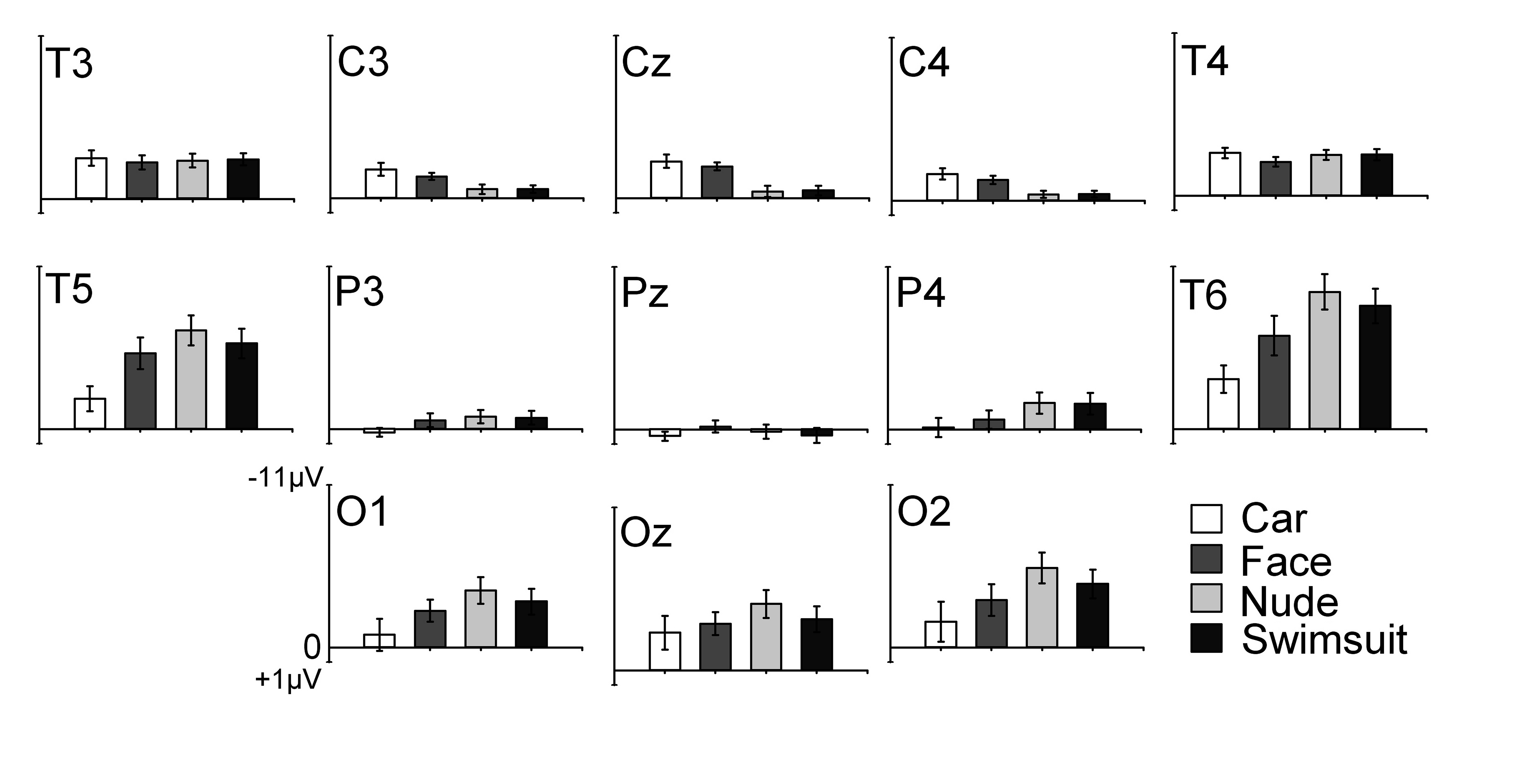

Supplement: Figure S1 — N170 amplitudes measured from all posterior recording channels in Experiment 1. The largest amplitudes for stimuli from each category were measured as follows (averaged across left and right channels): car, T3/T4, M = −2.8 µV; face, T5/T6 M = −5.7 µV; nude, T5/T6 M = −8.0 µV; swimsuit, T5/T6, M = −7.0 µV. (TIF) [file pone.0024408.s001.tif]

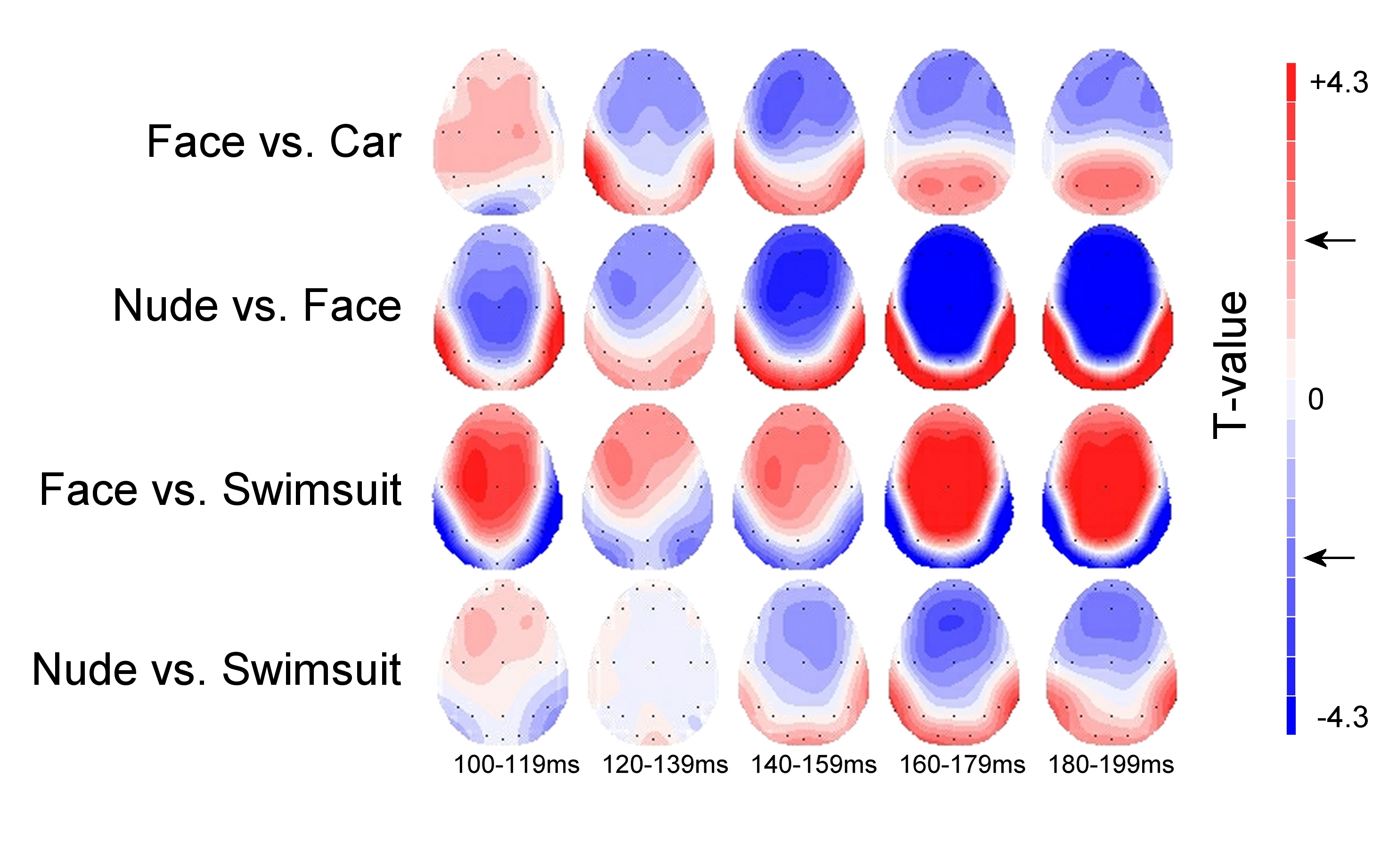

Supplement: Figure S2 — Statistical comparisons for the scalp topographies of the mean voltage amplitudes between face vs. car, nude bodies vs. face, face vs. swimsuit bodies, and nude bodies vs. swimsuit bodies in Experiment 1. The statistical comparisons are plotted in five consecutive 20-ms time windows starting at 100 ms post-stimulus. The arrows indicate color codes corresponding to the critical t-values (P<.05). (JPG) [file pone.0024408.s002.jpg]

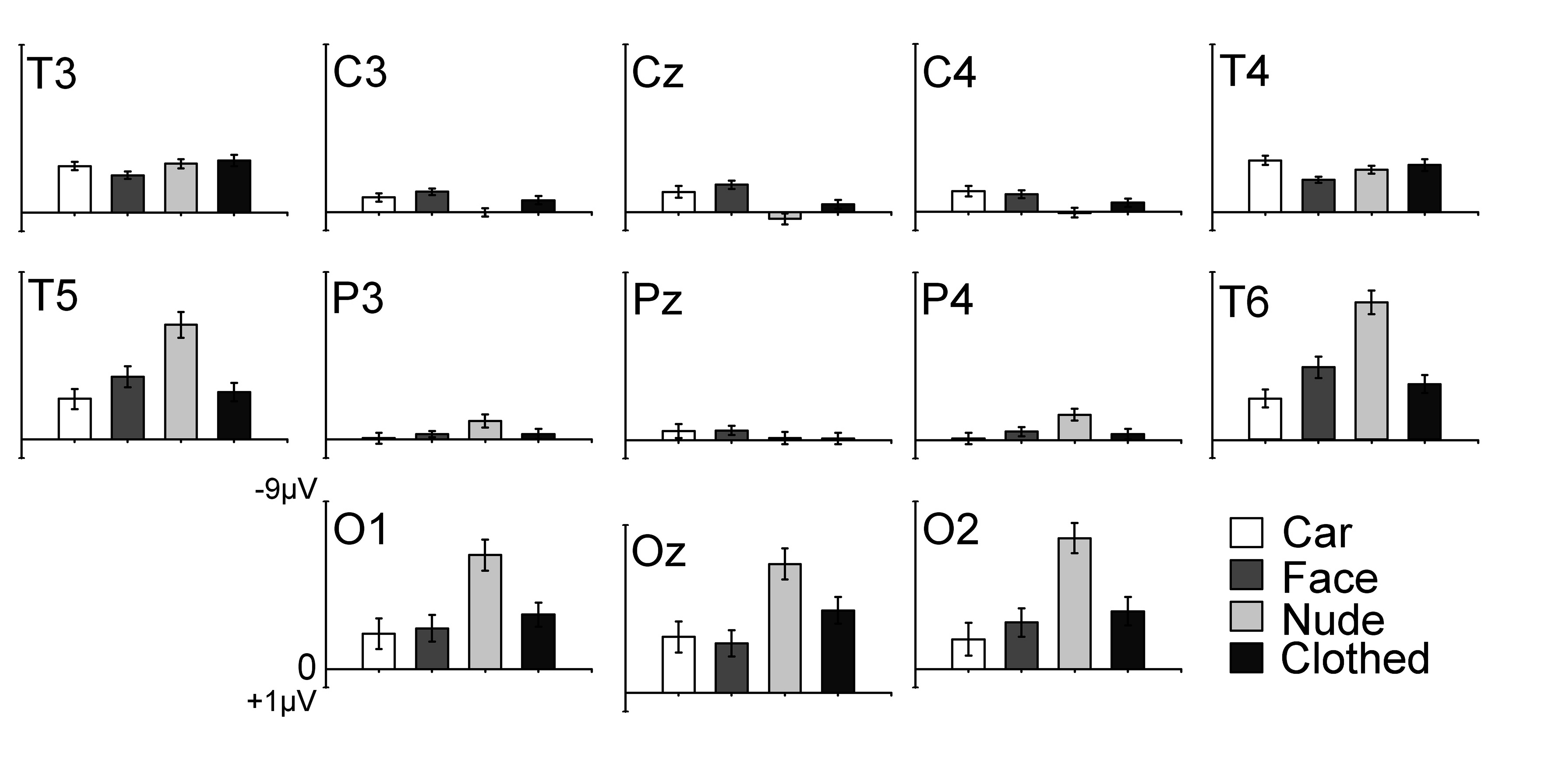

Supplement: Figure S3 — N170 amplitudes to intact versions of the stimuli measured from all posterior recording channels in Experiment 2. The largest amplitudes for stimuli from each category were measured as follows (averaged across left and right channels): car, T3/T4, M = −2.6 µV; face, T5/T6 M = −3.6 µV; nude, T5/T6 M = −6.7 µV; clothed, O1/O2, M = −3.0 µV. (TIF) [file pone.0024408.s003.tif]

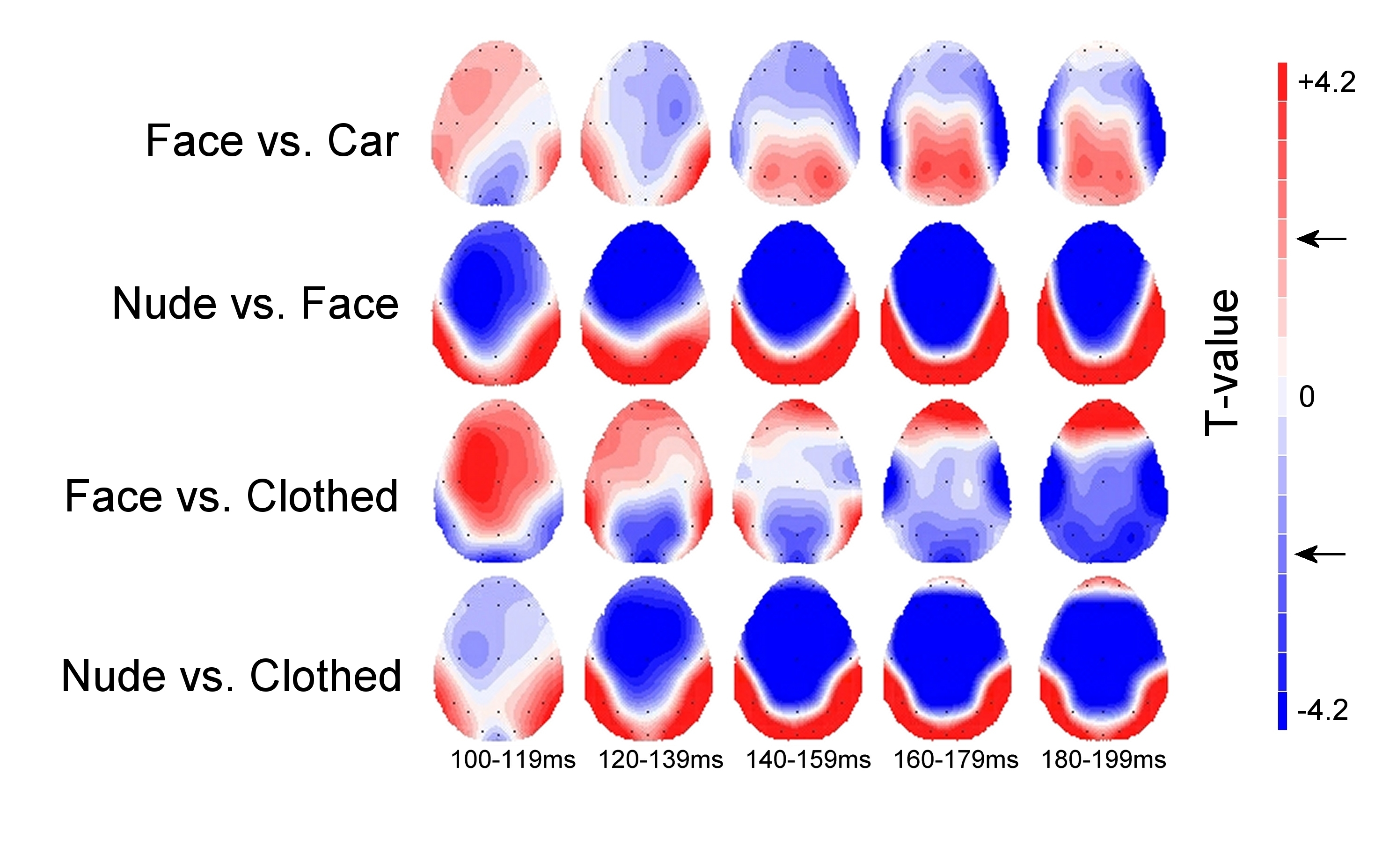

Supplement: Figure S4 — Statistical comparisons for the scalp topographies of the mean voltage amplitudes between face vs. car, nude bodies vs. face, face vs. clothed bodies, and nude bodies vs. clothed bodies in Experiment 2. The statistical comparisons are plotted in five consecutive 20-ms time windows starting at 100 ms post-stimulus. The arrows indicate color codes corresponding to the critical t-values (P<.05). (JPG) [file pone.0024408.s004.jpg]
